# Opposite effects of 5-HT/AKH and octopamine on the crop contractions in adult *Drosophila melanogaster*: Evidence of a double brain-gut serotonergic circuitry

**DOI:** 10.1371/journal.pone.0174172

**Published:** 2017-03-23

**Authors:** Paolo Solari, Nicholas Rivelli, Francescaelena De Rose, Lorenzo Picciau, Ludovico Murru, John G. Stoffolano, Anna Liscia

**Affiliations:** 1 Department of Biomedical Sciences, University of Cagliari, University Campus, S.P. 8, Monserrato (CA), Italy; 2 Stockbridge School of Agriculture, College of Natural Sciences, University of Massachusetts, Amherst, MA, United States of America; Wake Forest University, UNITED STATES

## Abstract

This study showed that in adult *Drosophila melanogaster*, the type of sugar—either present within the crop lumen or in the bathing solution of the crop—had no effect on crop muscle contraction. What is important, however, is the volume within the crop lumen. Electrophysiological recordings demonstrated that exogenous applications of serotonin on crop muscles increases both the amplitude and the frequency of crop contraction rate, while adipokinetic hormone mainly enhances the crop contraction frequency. Conversely, octopamine virtually silenced the overall crop activity. The present study reports for the first time an analysis of serotonin effects along the gut-brain axis in adult *D*. *melanogaster*. Injection of serotonin into the brain between the interocellar area shows that brain applications of serotonin decrease the frequency of crop activity. Based on our results, we propose that there are two different, opposite pathways for crop motility control governed by serotonin: excitatory when added in the abdomen (i.e., directly bathing the crop) and inhibitory when supplied within the brain (i.e., by injection). Finally, our results point to a double brain-gut serotonergic circuitry suggesting that not only the brain can affect gut functions, but the gut can also affect the central nervous system. On the basis of our results, and data in the literature, a possible mechanism for these two discrete serotonergic functions is suggested.

## Introduction

Neural control systems governing the regulation of meal size are subjects of great interest in both mammalian [1–4] and insect systems [5–8]. Research in two adult dipteran species, *Phormia regina* [9–11] and *Drosophila melanogaster* [12–17], has been particularly instructive in identifying the sensory, motor, and integrative components of the neural control systems for feeding. It is increasingly clear that multiple aminergic and peptidergic neuromodulators play an essential role in the operation of these control systems. In insects, reciprocal communication channels carry sensory information from gut to brain signaling feeding-induced gut distension [18–21] and information transmitted from brain to gut regulates muscle activity [22–24].

The initial designation of dipteran crop muscle pumps/sphincters for only *P*. *regina* were done by Thomson and Holling [25] and provide the basis of our current designation [26]. The present experiments provide further support for the generality of feeding control systems between insects and mammals [27,28]. Serotonin and octopamine were investigated as possible modulatory neurohormones based on a recent paper of Hsu and Bhandawat [29] on the organization of descending neurons in *Drosophila melanogaster*. The brain–gut axis is an essential part of the feeding control circuit in both mammals [2,30] and insects [8], especially when it comes to nutrient sensing [17,31].

The monoamine serotonin, or 5-hydroxytryptamine (5-HT), is one of the primary neurotransmitters modulating physiological and behavioral processes in the CNS and the serotonergic system is highly conserved in vertebrate species. Pioneering studies by Murdoch’s group [32] established the presence of serotonin in the ‘brain complex’, which included the subesophageal and supraesophageal ganglia, but excluded the optic lobes of adult *P*. *regina*, but made no mention of the neural plexus containing serotonin immunoreactive processes in the thoracico-abdominal ganglion (TAG) [10].

Literature exists on the complex interactions of neurotransmitters/neurohormones, such as serotonin, Phote-HrTH or *Phormia terraenovae* hypertrehalosemic hormone (i.e., adipokinetic hormone or AKH used in this paper), octopamine and the *Drosophila* insulin-like peptides (DILPs) of the insulin-producing cells (IPCs) in *D*. *melanogaster* [33–35], as they relate to sugar homeostasis; but, no data are available on the possible modulation of the crop muscle activity even though the crop is the major storage site for carbohydrates consumed by adult flies [36].

*Drosophila* is a well-known model organism, increasingly used in translational neuroscience and behavioral research [37,38]. Given the current interest on the gut-brain axis as a primary subject in the “start “of neurodegenerative disorders, such as Parkinson’s disease [39,40], it was evident that we needed to determine the possible modulatory effect of serotonin on crop contraction rate because in mammals it is known to modulate hypothalamic receptors, which control the size of carbohydrate-rich meals [41] and other aspects of ingestive behavior [42]. Thus, *D*. *melanogaster* possesses a complex serotonergic system featuring all major genes for 5-HT synthesis, metabolism and signaling [29] and provides a good model to test the effect of 5-HT on this important organ system.

Because of the importance of the adult crop organ in carbohydrate homeostasis (for a review see Stoffolano and Haselton [43]), we focused on providing supporting data showing the effect(s) for crop activity function in *Drosophila* based on an analysis of serotonin and octopamine along the gut-brain axis. At the same time, we tested the outcome of AKH treatment on crop activity according to its previously reported effect in *P*. *regina* [44]. Moreover, on the basis of our results, and of data in the literature, a possible mechanism regulating this modulation is discussed.

## Materials and methods

### Maintaining flies

Experiments in Italy were performed on 3–7 day old adult wild type (WT; Canton-S) *Drosophila melanogaster* males. After pupal emergence, adults were separated and reared on a standard cornmeal-yeast-agar medium in controlled environmental conditions (24–25°C; 60% RH; 12L/12D h) [45]. All flies emerging within 24 h were considered as one cohort. Insects were tested after being starved, but water satiated for 6 h in order to provide/ensure adults with empty crops. Preparations in which the crop was not completely empty were discarded.

In the U.S., mature, 3–7 day old males of *Drosophila* of the yw^1118^ strain were used for all bioassay experiments. Flies and standard cornmeal-sucrose-agar media were obtained from Dr. Michele Markstein’s lab at the University of Massachusetts in Amherst. During the 6 weeks of experimentation, flies were maintained in a MyTempMini Digital Incubator (H2200-HC) at 25°C on an ambient photoperiod.

### Solution preparation and reagents used in bioassay experiments

All solutions for the bioassay in the U.S. were prepared in double distilled water using Fisher or Sigma reagents of ACS grade or higher. According to preliminary experiments and data in literature [46,47], feeding solutions consisted of 100 mM glucose, fructose, or sucrose dissolved in distilled water and colored red for contrast by the addition of 25 mM amaranth dye. The bathing solution for dissection and physiological assay consisted of a *Drosophila* saline (NaCl 123 mM, KCl 2 mM, CaCl_2_ 1.8 mM, MgCl_2_ 8 mM, sucrose 35.5 mM and buffered at pH 7.1) [48], except for the experiments aimed at verifying the effects of the sugars on the crop contraction rate, that were performed in a sugar-free saline.

### Bioassay perfusion procedures

Adults were transferred from their stock vial and put into a fresh, clean vial of the same dimensions and secured with a wad of cotton packing material. Flies were then starved for 6 ±2 h to ensure complete emptying of the crop. Flies were cold anesthetized on crushed ice until all flies were immobilized. Flies were taken one by one and pinned to a 15 x 100 mm silicone lined petri dish (BioQuip 6187) using minuten pins (BioQuip Products, Inc.) through the upper left and right border of the thorax. Care was taken to avoid damaging any of the gastrointestinal tract. To prevent interference while feeding, the legs and wings of the subject were removed using two pairs of number 5, fine dissecting forceps (Dumont 11252–20). The fly was then fed the desired amount of feeding solution using a hand graduated 0.25 μL micro-capillary (Drummond 1-000-00025) to achieve incremental volumes as small as 63 nL by touching the solution to the fly’s proboscis.

Once fed the desired volume, and in order to prevent the fly from feeding on the bathing solution, the proboscis of the fly was carefully pinched shut and removed using forceps by pinching one forcep at the insertion of the proboscis and pulling from the body of the proboscis with the other pair always being careful not to rip off the entire head. The fly was then bathed in 100 μL of *Drosophila* saline. The upper lateral border of the abdomen was pinched with one pair of forceps, while the other pair grasped the cuticle of the abdomen and pulled across to tear open the abdomen, exposing the crop. The crop was allowed to adjust following the dissection for 3–4 min and then crop contractions were counted for 1 min. Contractions were counted by focusing on pump (P5) or the crop lobes. Contractions were tallied using a clicker at the start of each contraction. After the 1 min count, the fly was discarded and the procedure repeated with the next fly. In the event that a fly exhibited no contractions during the 1 min observation, it was removed as these flies likely represented errors in the procedure or outliers in their physiological state.

### Electrophysiological recordings of crop muscle activity from whole insects

**Fig 1:**
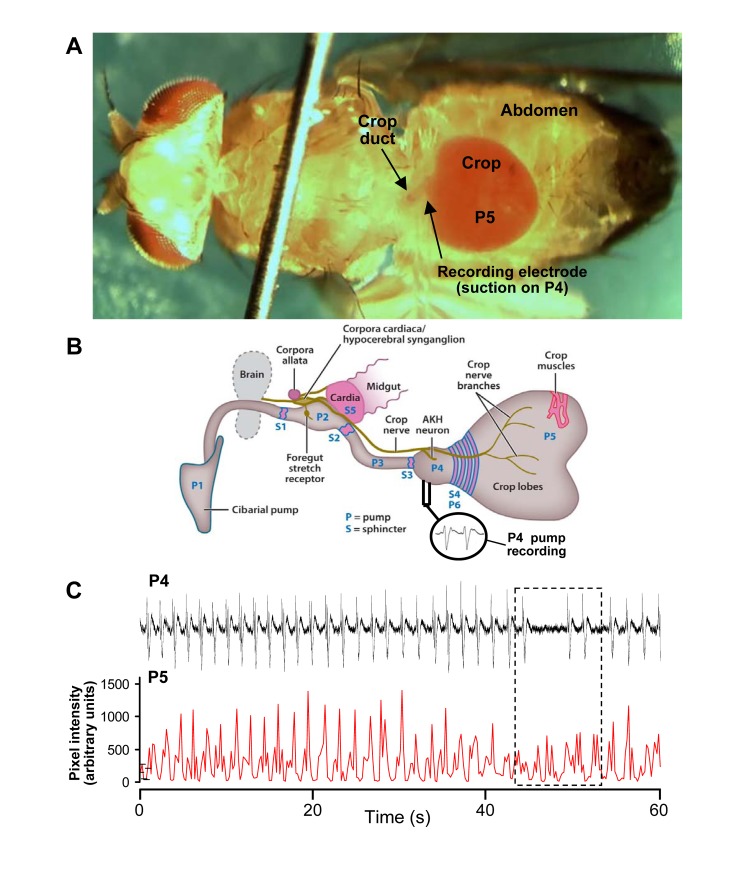
Crop preparation and recording of muscle activity. Recording layout of *D*. *melanogaster* crop activity (a) and schematic diagram of the typical dipteran foregut (b) showing various sphincters, pumps, crop nerve bundle and small segment of the muscles of the crop lobes. Shown in the circle area is a typical recording from a suction electrode from the crop P4. (c) Sample of a double simultaneous recording of crop muscle activity, showing the mAPs electrophysiologically recorded from P4 (upper trace), as compared to contractions of P5 (lower trace), that were determined by means of the Aviline software analysis [57]. Note that the two pumps can display either phase-locked or totally desynchronized (dashed rectangle) activities.

Flies were cold-immobilized at -20°C until they became inactive (typically 45–50 s), and then restrained in soft dental wax to limit movements, according to the procedure used by Liscia et al. [26]. A ventral section of cuticle was removed from the abdomen in order to expose pumps P5 for visualization of contractions and P4 for recordings (Fig 1). Dissections were performed using *Drosophila* saline. Extreme care was taken to ensure that the CNS, all peripheral nerves, and muscles remained intact; preparations that failed to resume crop activity after dissection were discarded. In these experimental situations, the labella of flies were also visible and accessible for feeding the fly and thereby inducing crop filling. Recordings of muscle activity (electromyograms, EMGs) from the surface of P4 were made *en passant*, by way of small borosilicate glass suction electrodes filled with *Drosophila* saline. The electrodes had a long shaft that ensured the necessary flexibility to follow small muscle contractions. With this experimental arrangement (Fig 1), stable recordings were possible for more than 2 h.

Recordings of muscle action potentials (mAPs) were preamplified and band-pass filtered (0.1–1000 Hz), using an A-M Systems (Everett, WA, USA) four-channel differential AC amplifier (Model 1700), digitized with an Axon Digidata 1344A A/D converter (sampling rate, 10 KHz per channel), and stored on a PC for further analyses (pClamp 10.0 software, Axon Instruments). During electrophysiological recordings, movements of P4 and P5 were monitored and video-recorded by way of a Moticam 2300 (3.0 M Pixel, USB 2.0) color digital camera coupled to the stereomicroscope (Zeiss, Stemi 2000-C); video information was stored on a computer as avi files and analysed with Motic Images Plus 2.0 ML software.

### Procedures and bathing solutions for testing the effects of exogenous compounds on crop activity

To determine if the rate of crop contractions depends on the type of sugar in the bathing medium, mAPs and contractions were recorded from P4 and P5, before and after replacing the saline bathing the crop with saline separately containing 0.1 M for each of glucose, fructose or trehalose that are carbohydrate molecules normally found in the hemolymph [49]. The 100 mM concentration for all sugars was chosen according to Miyamoto et al. [50] and also to our previous studies in the blowfly *Phormia regina* [44,51]. Prior to experimentation, flies were fed with 125 nL of a 100 mM sucrose aqueous solution added with 25 mM amaranth dye according to the bioassay results of the present study (i.e., a volume that allowed oscillations of crop activity in both decreasing and increasing directions).

The basal activity of the crop muscles was always recorded for at least 15 min before sugar-saline applications, and lasted for 30–45 min thereafter. The P4 mAPs and P5 contraction frequencies in the last minute of the basal activity were measured and compared to the activity recorded during the 1st minute after sugar-saline administration.

The same procedure was used to test the effects of the neuromodulators. Preliminary dose-response experiments (0.01 to 10 mM for 5-HT and octopamine and 0.001 to 1 mM for AKH) were performed on P5 crop pump in order to choose the proper drug concentration. According to these and previous [26,44,51] results, we used 1 mM 5-HT, 0.1 mM adipokinetic hormone (AKH, i.e. Phote-HrTH—*Phormia terraenovae* hypertrehalosemic hormone) and 1 mM octopamine. Moreover, according to data in literature [52–54], in order to tentatively classify the 5-HT receptor on the crop muscles, the selective antagonist of 5-HT2 receptors ketanserin was tested at 1 mM on both crop pumps. All counts include interburst intervals when present. All chemicals used in the present study were obtained from Sigma-Aldrich. Fresh solutions of sugars, neuromodulators and saline were prepared daily.

Solutions were applied, by way of a microinjection system (Narishige, IM-4B), by replacing a 5 μl drop of solution removed from the preparations with a new 5 μl drop of solution. Thus, each new saline, sugar- or neuromodulator-saline application always occurred after removal of the hemolymph or any previous tested solution. Prior to solution administration, the basal activity in the presence of 5 μl of freshly-applied saline was also tested in order to exclude artifacts and/or ‘‘non-specific” variation in crop activity due to the removal and addition of a drop of liquid.

As for the evaluation of the 5-HT effects along the gut-brain axis in *D*. *melanogaster*, brain injections of 5-HT and control saline were made using a glass micropipette (tip diameter 0.5 mm) by means of a a small incision into the head capsule between the interocellar setae, according to Kuebler and Tanouye [55].

### “Cropograms”

Contractions of P5 were recorded using video-microscopy followed by computer analysis of the movements from frame to frame according to the procedure adopted by Middleton et al. [56]. This approach produces a “cropogram” in which the contractions at several sites on the same preparation can be recorded and compared. The video recordings were converted to a resolution of 640 × 480 pixels, at 5 frames/sec (300 frames/min), so that each frame could account for the instantaneous “contraction state” of the crop muscles at 200 msec intervals. Each video was analysed using a custom program [57] while the computer mouse was used to overlay lines on the video frames so that each line crossed the light/dark boundary between the crop and the background. For each frame, the distance of the light/dark interface from the start of the line was determined, along with the mean squared difference in intensity between successive frames of the pixels along the line (Fig 1C). This recorded the displacement in the plane of focus, but any movement in the vertical direction was not measured. Data were saved in a Microsoft Excel format and mean peak height and intervals between peaks calculated. In this way the amplitude of the crop contractions were evaluated before and after supply of neuromodulators.

### Statistical analysis

Statistical analysis for the bioassay was performed using JMP version 12. To test the factorial design of the experiment, a one-way and two-way (factorial) ANOVA was used and followed by a Tukey-Kramer t-test. Repeated-measures ANOVA was used to evaluate the effects of perfusion applications of exogenous sugars on crop P4 and P5 contraction rate, while the effects of neuromodulators on both contraction frequency and amplitude were estimated by way of a one-way ANOVA analysis. Repeated-measures ANOVA was also used to assess significant differences for crop P4 and P5 activity following injection of 5-HT into the brain vs. perfusion application directly onto the crop. The latter ANOVA analyses were performed using Statistica for Windows (version 7.0; StatSoft, Tulsa, OK, USA). Post-hoc comparisons were conducted with the Tukey test and *P* values *<* 0.05 were considered significant.

## Results

### Effect of sugar-type within the crop and crop volume on P5 contraction rate using a bioassay

**Fig 2:**
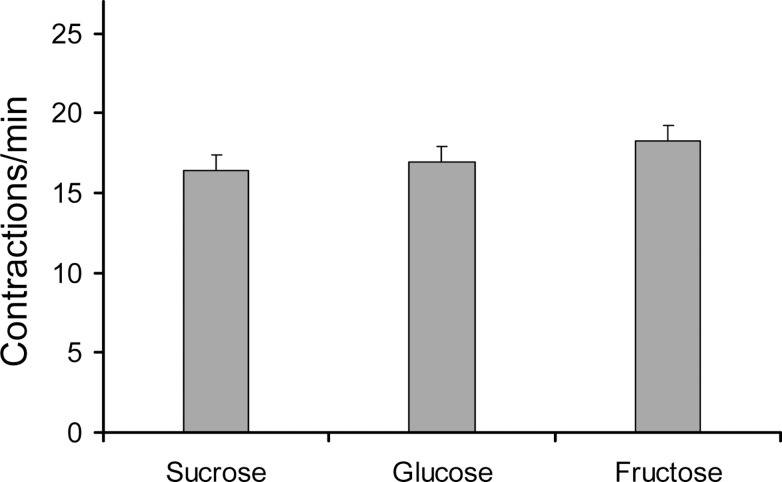
Effect of various sugars ingested and their influence on crop contraction rate in adult *D*. *melanogaster*. Mean values ± SE (vertical bars) from 20 insects (yw-1118 strain) for each sugar tested. No significant differences were detected amongst the sugars ingested (P > 0.05; Tukey-Kramer test subsequent to one-way ANOVA).

Separately feeding the flies different sugars showed that the mean rate of contraction was 16.95 for sucrose, 16.40 for glucose and 18.25 cont/min for fructose and therefore that the type of sugar ingested had no effect on crop contractions (Fig 2). The differences of the means using a one-way analysis of variance (one-way ANOVA) revealed an F ratio of 0.1887 and a p-value of 0.8286 suggesting that there was no significant difference between the type of sugar tested and crop contraction rate. A Tukey-Kramer test was also run. P-values of 0.8217 for the fructose-sucrose comparison, 0.9074 for the fructose-glucose comparison, and 0.9827 for the glucose-sucrose comparison confirmed that there was little difference between the test groups.

**Fig 3:**
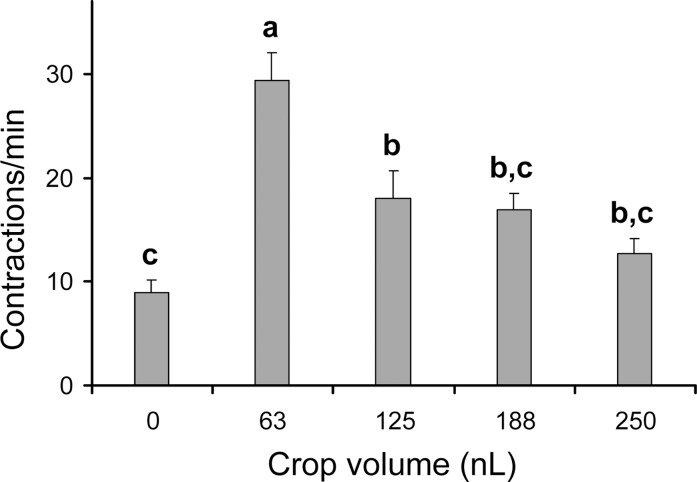
Effect of crop volume on contraction rate in adult D. melanogaster. Mean values ± SE (vertical bars) from 12 insects (yw-1118 strain) for each volume tested. Bars followed by different letters are significantly different (P < 0.05; Tukey-Kramer test subsequent to one-way ANOVA).

Data was also analyzed according to crop volume, irrespective of sugar type, to examine the effect of volume on crop contraction rate (Fig 3). The mean rate of contraction was 9.00 cont/min for the 0 nL group and increased significantly to 29.33 for the 63 nL group. The mean rate then declined steadily to 18.00, 16.91, and 12.75 cont/min for the 125, 188, and 250 nL groups, respectively. This difference was verified by a one-way ANOVA that revealed an F ratio of 14.5222 and a p-value less than 0.0001 indicating significant difference between one or more of the volume groups. To determine which groups were significantly different, a Tukey-Kramer test was performed. The results showed significant differences (p < 0.001) between all groups and the 63 nL group, as well as a significant difference between the 125 nL and 0 nL groups.

To ensure that there was no difference between sugar and volume ingested, a two-way, or factorial ANOVA was run. The results of this test were similar, although not identical to the individual one-way ANOVAs, with a p-value of 0.6971 for the comparison of sugars, and a p-value < 0.0001 for the comparison of volumes. A final set of one-way analyses of contraction rate by sugar for each volume and by volume for each sugar revealed comparable levels of significance to earlier tests.

### Effects of perfusion applications of exogenous sugars on crop contraction rate using electrophysiology

**Fig 4:**
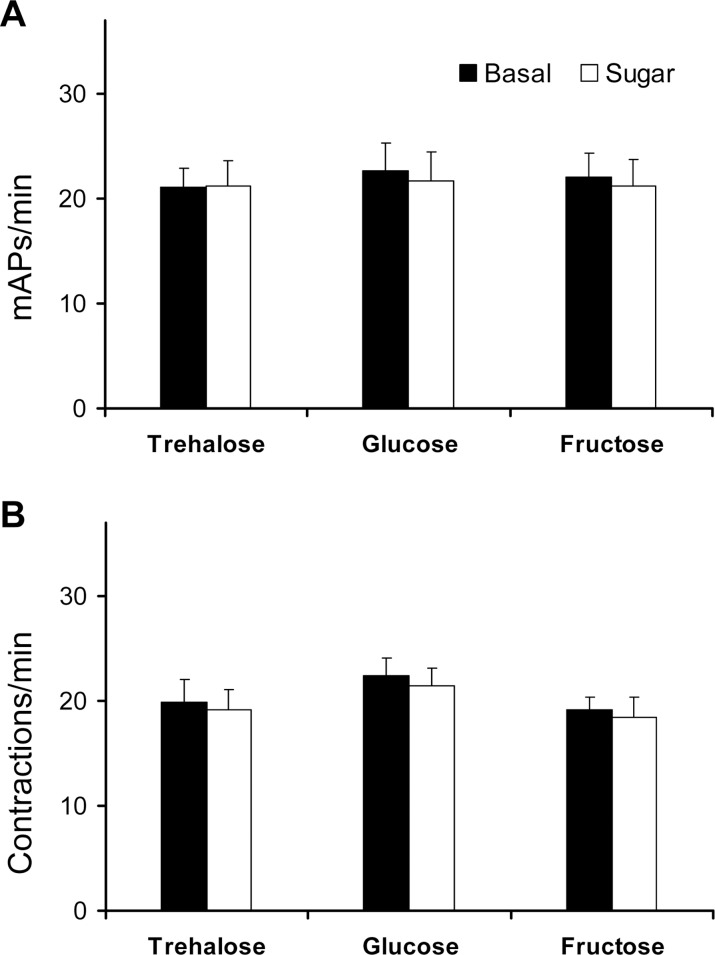
Effect of various sugars bathing the crop and their influence on crop contraction rate in adult *D*. *melanogaster*. Frequency of mPAs electrophysiologically recorded from P4 (a) and of contractions visually recorded from P5 (b) of *Drosophila* Canton-S strain, determined over a 1-min interval, following replacement of hemolymph with 0.1 M trehalose, glucose or fructose with respect to *Drosophila* saline (basal activity). Mean values ± SE (vertical bars) from 8 (trehalose and glucose) or 7 (fructose) replicates in (a) and from 18 (trehalose and 20 (glucose and fructose) replicates in (b). No significant differences were detected among sugars for both P4 and P5 (P > 0.05; Tukey test subsequent to one-way ANOVA).

The mean mAP frequency, electrophysiologically recorded from P4, and the contraction frequency visually detected from P5 are represented in Fig 4A and 4B, before (basal activity) and after perfusion applications on the crop of the different sugar solutions (100 mM of each trehalose, glucose or fructose dissolved in saline). None of the sugar solutions affected the basal activity of P4 (Fig 4A), regardless of the sugar tested, trehalose (from 21.1 ± 1.7 to 21.2 ± 2.5 mAPs/min; F_[1,7]_ = 2.1425, p = 0.19), glucose (22.6 ± 2.6 to 21.7 ± 2.8 mAPs/min; F_[1,7]_ = 0.3357, p = 0.58), or fructose (22.1 ± 2.3 to 21.2 ± 2.6 mAPs/min; F_[1,6]_ = 0.8532, p = 0.39).

All tested sugars were also ineffective on P5 contraction rates (Fig 4B), irrespective of the sugar tested, trehalose (from 19.8 ± 2.2 to 19.2 ± 1.9 contractions/min; F_[__1__,__17__]_ = 0.0914, p = 0.77), glucose (from 22.4 ± 1.7 to 21.5 ± 1.6 contractions/min; F_[1,19]_ = 0.4811, p = 0.49), or fructose (from 19.15 ±1.2 to 18.5 ± 1.8 contractions/min; F_[1,19]_ = 0.0791, p = 0.78). Therefore, both P4 and P5 contraction rates are not affected by the perfusion applications of 100 mM sugar solutions directly onto the crop.

### 5-HT and AKH enhance, while octopamine and ketanserin depress the crop pumps contraction rate

**Fig 5:**
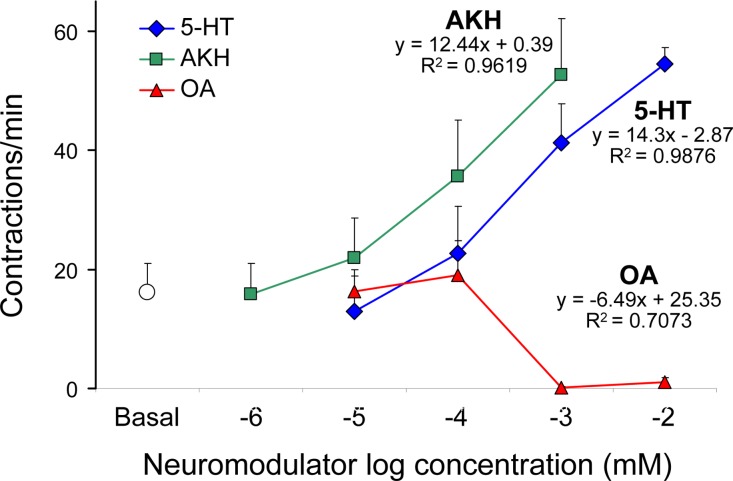
Dose-response curves for various neuromodulators on P5 crop contraction rate. Frequency of contractions visually recorded from P5 of *Drosophila* Canton-S strain, determined over a 1-min interval, following replacement of hemolymph with 0.01–10 mM 5-HT and octopamine (OA) and 0.001–1 mM AKH, as compared to the activity in *Drosophila* saline (basal). Mean values ± SE (vertical bars) from 10 replicates for each compound.

On the basis of results shown in Fig 5, a direct dose-response relationship was found for 5-HT and AKH, while inverse for octopamine on the P5 crop muscle activity. These and previous [26,44,51] results allowed us to select the 1 mM concentration for 5-HT and octopamine and 0.1 mM for AKH for further experiments.

**Fig 6:**
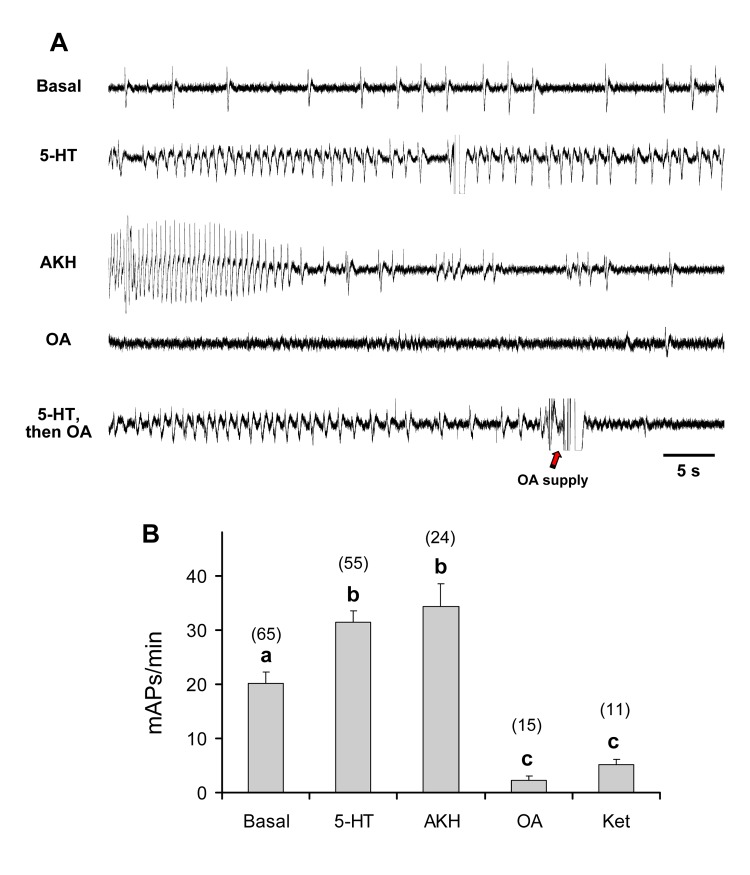
Effect of various neuromodulators on crop contraction rate in adult *D*. *melanogaster*. Samples of electromyograms (a) and frequency of mPAs (b) recorded from P4 in *Drosophila* Canton-S strain, determined over a 1-min interval, following replacement of hemolymph with 1 mM 5-HT, octopamine (OA) and ketanserin (ket) and 0.1 mM AKH, as compared to the activity in *Drosophila* saline (basal). Mean values ± SE (vertical bars); number of replicates for each compound is indicated in brackets. Bars followed by different letters are significantly different (P < 0.05; Tukey test subsequent to one-way ANOVA).

The neuromodulators tested on P4 and P5 significantly affected the muscle activity of both P4 (F_[4,165]_ = 17.128, p < 0.0001) and P5 (F_[4,184]_ = 28.130, p < 0.0001). Comparisons showed that the basal activity, electrophysiologically recorded, from P4 (20.1 ± 2.2 mAPs/min; Fig 6A and 6B) was greatly enhanced to a similar extent by both 5-HT and AKH (31.4 ± 2.1 and 34.3 ± 4.1 mAPs/min, respectively). Conversely, the addition of octopamine and the 5-HT antagonist ketanserin strongly depressed the activity of P4 to 2.3 ± 0.7 and 5.1 ± 1.0 mAPs/min, respectively.

**Fig 7:**
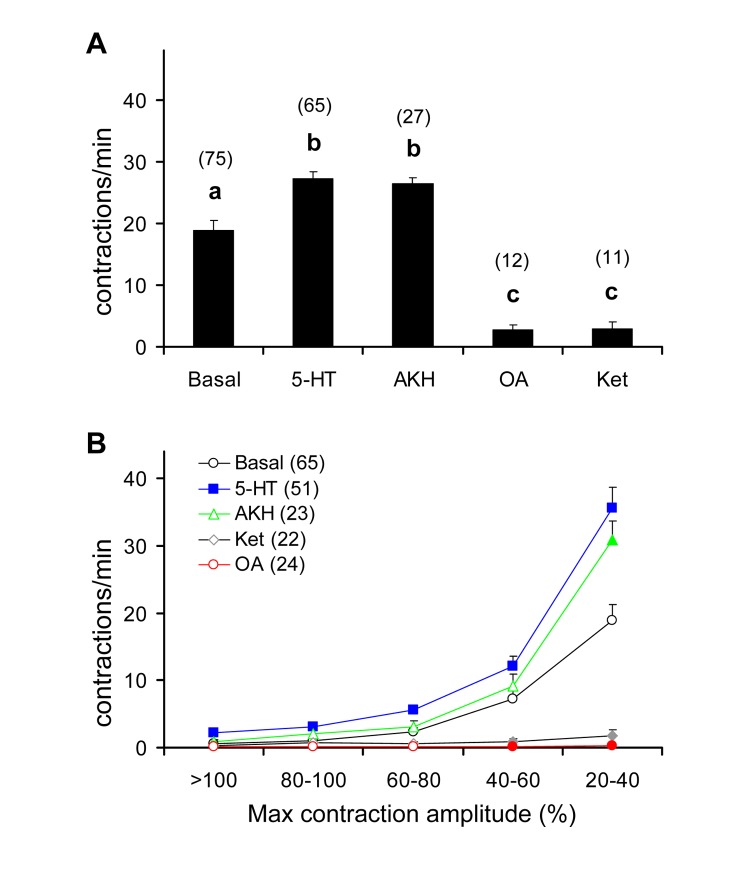
Effect of various neuromodulators on crop contraction rate in adult *D*. *melanogaster*. (a) Frequency of contractions visually recorded from P5 in *Drosophila* Canton-S strain, determined over a 1-min interval, following replacement of hemolymph with 1 mM 5-HT, octopamine (OA) and ketanserin (ket) and 0.1 mM AKH, as compared to the activity in *Drosophila* saline (basal). Mean values ± SE (vertical bars); number of replicates for each compound is indicated in brackets. Bars followed by different letters are significantly different (P < 0.05; Tukey test subsequent to one-way ANOVA). (b) Frequency of P5 contractions in the same Canton-S strain, classified on the basis of the percentage of maximal contraction amplitude elicited, as assessed by the Aviline software analysis [57]. Mean values ± SE (vertical bars); number of replicates for each compound is indicated in brackets. Filled symbols represent significant differences with respect to the basal level (P < 0.05; Tukey test subsequent to one-way ANOVA).

The same results were also obtained for P5, whose basal activity visually recorded (18.8 ± 1.6 contractions/min; Fig 7A and S1 Movie) was enhanced by 5-HT and AKH, while diminished by octopamine and ketanserin (Fig 7A and S2 and S3 Movies) in a magnitude order similar to that detected in the case of P4. This suggests that the two crop pumps share comparable sensitivity to the tested neuromodulators, at least in terms of muscle action potential and contraction frequency.

Interestingly, 5-HT was also found to enhance, with respect to the basal level, the number of P5 contractions at any class of contraction amplitude considered (Fig 7B), with a peak at the 20–40% amplitude class (from 18.9 ± 2.4 to 35.6 ± 3.0 contractions/min) (i.e., the one comprising the lowest contraction amplitudes). Conversely, AKH enhanced the number of P5 contractions only within the 20–40% class of contraction amplitude to 30.8 ± 2.9 cont/min. Contrary to 5-HT, AKH did not affect the number of contractions at higher amplitudes. Octopamine and ketanserin virtually silenced the P5 activity and in no case elicited a number of contractions with amplitudes higher than those of the basal activity. Therefore, both 5-HT and AKH are able to enhance the P5 contraction frequency, but 5-HT also acts by increasing the contraction amplitude of this pump at a higher extent than AKH.

### Opposite effects of 5-HT injected into the brain vs. perfusion application on the crop

**Fig 8:**
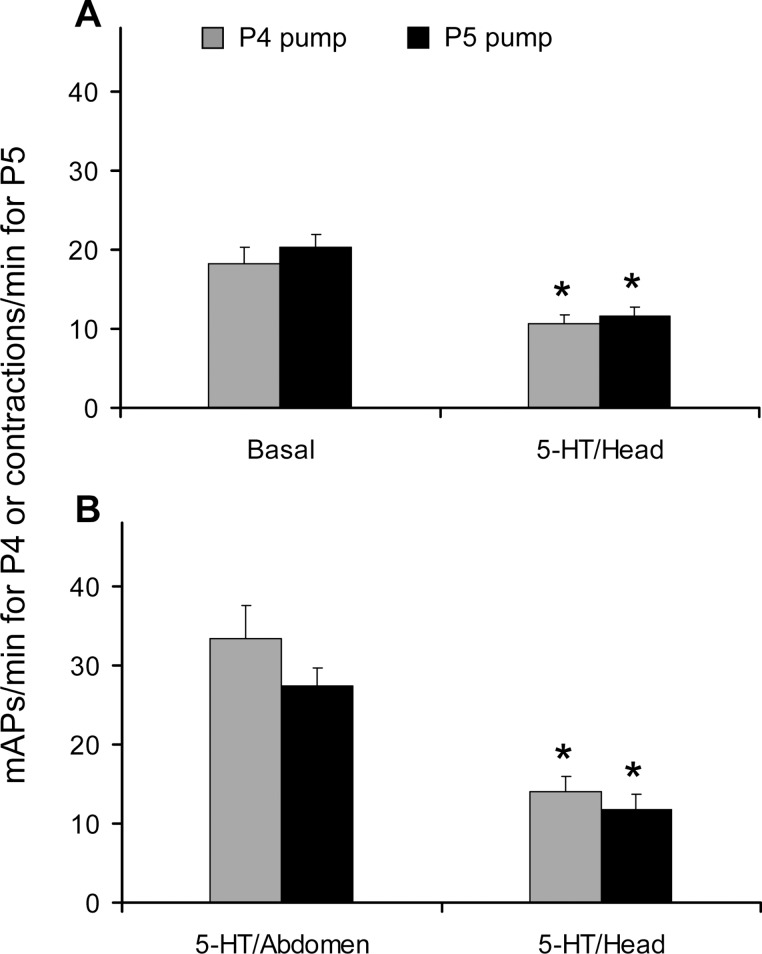
Opposite effects of 5-HT injected into the brain *vs*. perfusion application on the crop. Frequency of mPAs, electrophysiologically recorded from P4 and of contractions visually recorded from P5 in *Drosophila* Canton-S strain, determined over a 1-min interval, following injection of 5-HT into the brain as compared to the activity in *Drosophila* saline (basal) (a) or to the activity previously enhanced by perfusion application of 5-HT into the abdomen. Mean values ± SE (vertical bars) from 15 replicates in (a) and 16 replicates in (b). The asterisk indicates a significant difference (P < 0.05; Tukey test subsequent to repeated-measures ANOVA) with respect to the basal level.

Injections of 5-HT into the brain of the fruit fly significantly affected the basal muscle activity of both P4 (F_[1,14]_ = 20.391, p = 0.0005) and P5 (F_[1,14]_ = 26.077, p = 0.0002) (Fig 8A). Comparisons of the data showed that the basal activity, electrophysiologically recorded, from P4 was significantly diminished by 5-HT injections from 18.3 ± 1.9 to 10.6 ± 1.1 mAPs, while the basal contraction frequency visually recorded for P5 was reduced from 20.3 ± 1.6 to 11.5 ± 1.2 cont/min, thus confirming that in *Drosophila* there is no statistical difference in the frequency between the two pumps.

Injections of 5-HT into the brain—while recording from the crop—decreased crop contraction rate even after its activity was previously enhanced by perfusion application of 5-HT the abdomen (Fig 8B). This occurred for both P4 (F_[1,15]_ = 20.878, p = 0.0004) and P5 (F_[1,15]_ = 23.083, p = 0.0002). 5-HT-induced crop activity was significantly diminished by brain applications of 5-HT from 33.4 ± 4.2 to 14.1 ± 1.9 mAPs/min in the case of P4 and from 27.3 ± 2.3 to 11.8 ± 1.9 cont/min for P5. Therefore, data shows that the two pumps respond in a similar fashion to brain applications of 5-HT and that two different, opposite pathways for crop motility control are likely to exist for 5-HT: inhibitory when supplied within the brain, excitatory when added in the abdomen in order to directly bathe the crop. It is to be pointed out that preliminary experiments showed that control saline injections significantly altered neither the P4 nor the P5 muscle activity (results not shown).

## Discussion and conclusions

### Crop volume, but not the sugar type, affects the crop contraction rate

Unlike the information available for the crop of *P*. *regina* [11], very little information exists on the detailed structure of the crop of adult *D*. *melanogaster* or, in fact, any other Diptera [43]. Even though the crop is essential for survival/reproduction, the details of the dipteran crop, such as the specific pumps and sphincters, have only been determined for *P*. *regina* [58]. Thomson used behavioral feeding experiments to determine the various regions of the crop while in this study, we used electrophysiological recordings to demonstrate the presence of two pumps (i.e., P4 and P5), which are very similar to those of *P*. *regina* in response to various treatment, but might vary as to their numbering once the *Drosophila* crop system (i.e., sphincters/pumps) has been determined (see Fig 1B).

*P*. *regina* adults can take up to 18 μl (18,000 nL) in their crop while *D*. *melanogaster*, which is much smaller and in this study, imbibed only up to 250 nL or 72X less the amount of *P*. *regina*. Males of *P*. *regina* weigh an average of 43.67 mg while *D*. *melanogaster* males only weigh about 0.99 mg [59]. Our results for volumes consumed by *D*. *melanogaster* (maximum of 250 nL) are within the range of consumption volumes shown for the same species by Edgecomb et al. [60] and average consumption by adults of 150 nL fed a 5% sucrose solution [13].

Using the feeding procedures described above, we generated Fig 3, which shows the effect of crop volume on crop contraction rate. As with *P*. *regina* [61] and *Musca domestica* [62], the trend is the same (i.e., empty crops have few contractions; and, as crop volume increases there is an increase which peaks, followed by a decline where a full crop has contraction rates similar to those with the least volume). This decline is believed to be due to the inability of the supercontractile muscles to function beyond a confined stretch. If crop contractions, or the contraction of specific pumps (especially P4), are essential to move fluids out of the crop and into the midgut, one could ask how this is accomplished. Since the crop is a storage organ for sugars, and in other dipteran such as *P*. *regina* the type of sugar bathing the crop was found to modulate the crop contraction rate [51,61], we tested the effect of different sugar types. Based on these result, we showed that in adult *Drosophila melanogaster*, the type of sugar—either present within the crop lumen or in the bathing solution of the crop—had no effect on crop muscle contraction, which differed from *P*. *regina*, thus other mechanisms are mainly involved in the crop contraction such as the volume, as above discussed, and as the major aim of our study, the possible involvement of neurotransmitters/neuromodulators.

### 5-HT and AKH enhance, while octopamine and ketanserin depress the activity of the crop pumps

We have demonstrated, using adult *D*. *melanogaster* that, like in *P*. *regina* [11,26,51], the foregut musculature of the crop is stimulated by the hemolymph borne 5-HT and AKH, even if to a different extent. In *Drosophila*, there are seven insulin-like peptides (DILP1–7), three of which (DILP2, 3 and 5) are produced in median neurosecretory cells of the brain, designated IPCs, that have been reported to be regulated by serotonergic neurons [63].

The biogenic amine octopamine is another neurotransmitter reported to be involved in the IPC activity in *Drosophila* [33]. An interaction exists between IPC products that project to the corpora cardiaca whose intrinsic cells produce AKH, thus the DILP-AKH system of *Drosophila* and the insulin-glucagon system of mammals may have analogous roles in regulating sugar homeostasis and metabolism [64].

Our results show that in *Drosophila* exogenous applications of 5-HT on crop muscles increases both the amplitude and the frequency of the crop contraction rate, while AKH mainly enhances the crop contraction frequency. Conversely, octopamine virtually silenced the overall crop activity. Moreover, we report for the first time an analysis of serotonin effects along the gut-brain axis in *D*. *melanogaster*. Our results show that the brain applications of 5-HT decrease the frequency of both P4 and P5 pumps.

Therefore, two different, opposite pathways for crop motility control are likely to exist for 5-HT: excitatory when added in the abdomen in order to directly bathe the crop and inhibitory when supplied within the brain. As for the latter, Nässel et al. [34] suggested that the 5-HT1A receptor (i.e., in the brain) is indeed inhibitory and inhibits both adenylate cyclase (AC) and protein kinase A (PKA) and subsequently inactivates the cAMP response element binding protein (CREB) (see Nichols and Nichols [65]). Activated CREB is known to inhibit insulin signaling [66], and therefore inhibition of AC, PKA, and CREB would stimulate insulin signaling, while octopamine activates the AC and PKA. Adult IPCs display cell-autonomous sensing of circulating glucose, coupled to evolutionarily conserved mechanisms for DILP release. The glucose-mediated DILP secretion is modulated by neurotransmitters and neuropeptides, as well as by factors released from the intestine and adipocytes. In this respect, an important significance is dedicated to the 5-HT role in mediating important behaviors in mammalian systems, which range from feeding, aggression, and sleep, to cognition. *Drosophila* adults express four homologs of mammalian 5-HT receptors: the 5-HT1A, 5-HT1B, 5-HT2, and 5-HT7 receptors. All of which are G-protein coupled receptors. 5-HT1A and 5-HT1B, which are known to inhibit adenylate cyclase while 5-HT7 stimulates it, whereas the mechanism of 5-HT2 activation was showed by means of the 5-HT2 receptor antagonist, ketanserin resulting in an increase of IP3 and diacylglycerol [35,54,63,65] and similarly to what reported for the gut of the lepidopteran caterpillar *Spodoptera frugiperda* [52] or the heart and aorta in *D*. *melanogaster* larvae [53].

Based on the above studies, we found that according to its antagonist role, ketanserin has a dramatic effect on significantly reducing muscle activity of both pumps P4 and P5, thus confirming that serotonin directly affects these muscles other than acting via a brain-gut axis, as assessed by the fact that also the activity of an isolated crop is enhanced by 5-HT supply (S4 Movie); and, thus it is likely enhancing Ca^2+^ release from the intracellular stores via IP_3_ pathway. Moreover, exogenous abdominal application of 5-HT could act as in *Phormia* [26,51], by enhancing the calcium entry into the cell, followed by activation of a Ca2+-activated K+ conductance (Ik(Ca)), which sustains the mAP repolarization phase in such a way that further mAPs can be generated early and the frequency of P4 and P5 increased. Alternatively, as shown in other invertebrates [67], 5-HT might involve a cAMP dependent pathway leading to phosphorilation, thus resulting in a change in input resistance of the muscle by closing particular channels.

In *Drosophila*, AKH, similarly as 5-HT, enhanced both pumps activity, while in *Phormia* it was demonstrated that there was an increasing effect in P4, but a decreasing effect in P5 [44]. It has been showed that AKH is released by the intrinsic cells of the corpora cardiaca (CC) [68], which are affected by DILPs [35]. There is no doubt as to the importance of the DILP-AKH system in regulating the metabolism in *Drosophila*, which is similar to what happens in the insulin–glucagon system of mammals (see for a review Bednářová et al. [69]). As AKH released from CC can also have a neuro-modulatory effect on the central nervous system, more investigation is needed to better elucidate the possible mechanism underlying the AKH effects along the gut-brain axis in *Drosophila*.

One study that needs further verification, however, is that of Audsley et al. [70] where they report in adult *D*. *suzukii* that AKH was found only in the corpora cardiaca and was not found in the nerve bundle going to the crop. This is contrary to the reports of Lee and Park [68] on *D*. *melanogaster* and that of Stoffolano et al. [44] for *P*. *regina*.

The results of this study suggest an opposite effect of the neuromodulator octopamine between *Drosophila* and *Phormia*. In fact, in *Drosophila* it silenced both the basal and the 5-HT- and AKH-induced crop contraction rate, while in *Phormia* an increase in feeding behavior after octopamine supply was detected [71]. In this respect, a direct correlation between hyperphagia to sugars and clonidine, an agonist of the octopamine receptor, was demonstrated by Long and Murdock [71], as well as enhancing sugar feeding [72]. As for the possible mechanism underlying the direct octopamine effects on the crop, it was suggested that octopamine plays a role in activating IPCs by increasing their activity, but it is not insulin-dependent [33]. Our results indicate the presence of octopamine receptors on the crop muscle, whose activation leads to a total block of the crop contraction, acting on both the frequency and amplitude, likely counteracting with the Ca^2+^ pathway. As a general consideration, it is known that dromyosuppressin, 5-HT and possibly other neuromodulators and chemicals (Table 1) are involved in different flies in regulating food intake and expulsion from the crop to the midgut for digestion or for regurgitation (aka bubbling behavior [43]).

**Table 1:** Chemicals affecting crop muscle contractions in adult flies. Species: 1-*D*. *melanogaster*; 2-*P*. *regina;* 3-*M*. *domestica*; Pumps/Valves based on Thomson [61].

Chemical	Effect on crop	Species	Pumps/Valves	References
Dromyosuppressin (DMS), TDVDHVFLRFamide	Negatively modulates contractions	1, 2	Lobes	[76–80]
Benzothonium (Bztc)	Mimics the myosuppressin effect of DMS	2, 3	Lobes	[80,81]
Serotonin (5-HT)	Increases activity of P4	2	P4	[26]
Drosulfakinins, drosulfakinin 0 (DSK 0)	Decreases contractions	1	Lobes	[82]
TPAEDFMRFamide	Decreases contractions	1	Lobes	[76,77,83]
AKH or Phote-HrTH (*P*. *terraenovae* hypertrehalosemic hormone)	Negatively modulates P4, but increases P5	2	P4, P5	[44]
Spider peptide toxin- GsMTx-4	Negatively modulates P5	2	P5	[11]
Trehalose	Increases P5 contractions	2	Lobes	[51,61]
Glucose	Reduces P5 contractions	2	Lobes	[51,61]
	Decreases rate of opening of V2, i.e. it requires more peristaltic waves in P3 to cause V2 to open	2	V2/P3	[58]
CavTachykinin, Callitachykinin-1	Increases rate of contractions V4/P6 but alters P5	2	V4/P6	[84]
Peptide flatline (FLT)	Reduces the number of lobe contractions	1	Lobes	[85]

### Concluding remarks

Finally, it is now universally accepted that *Drosophila* is a well-known model organism currently being used in translational neuroscience and behavioral research [37,38]. Given the current interest on the gut-brain axis as a primary subject in the “start”of neurodegenerative disorders, such as Parkinson’s disease [39,40,73] and also the importance of microbiota in the gut-brain axis and serotonergic control [74], our research focused on and provides new information on crop contraction mechanisms (as a part of the gut) and the serotonergic control in this fly. In particular, our results point to a double brain-gut serotonergic circuitry, thus suggesting that not only the brain can affect gut functions, but the gut can also affect the central nervous system.

It is not surprising that there is evidence of nervous, or neuroendocrine control (see for a review Lemaitre and Miguel-Aliaga [75]), rather than via hemolymph molecules, on this important dipteran storage organ. This is evident by the numerous reports listed in Table 2, regarding the presence of a neural bundle [11], which houses several axons going to the crop in adult *Drosophila* and other insect species. The trafficking direction of various chemicals between those stored and/or produced within the CC and those of the crop are essential in order to understand the gut-brain axis of this important organ system.

**Table 2:** Reports using various techniques (microscopy, antibodies or molecular probes, liquid chromatography (LC) and mass spectrometry (MS)) showing the nerve going to the crop and in some cases its content.

Technique used	Species	References
A. Microscopy (light, TEM, SEM)		
Light microscopy/dissection	mosquito	[86]
	*Calliphora erythrocephala*	[87]
	*Glossina morsitans*	[88]
	*Glossina brevipalpis*	[89]
*SEM and TEM*	*Phormia regina*	[11]
B. Immunostaining		
Insulin-like peptide	*Drosophila melanogaster*	[90]
	*Aedes aegypti*	[91]
Serotonin	*Stomoxys calcitrans*	[92]
Anti-AKH antibody	*D*. *melanogaster*	[68]
C. Molecular, LC + MS		
Ilp2-Gal4	*D*. *melanogaster*	[93]
Diuretic hormone 44 (Dh44),	*D*. *melanogaster*	[94]
Myosuppressin	*Drosophila suzukii*	[70]

## Supporting information

S1 Movie Basal crop contraction rate in adult *D*. *melanogaster*. (WMV)

S2 Movie Enhancing effect of 5-HT on crop contraction rate in adult *D*. *melanogaster*. Following replacement of hemolymph with 1 mM 5-HT.
(WMV)

S3 Movie Diminishing effect of ketanserin on crop contraction rate in adult *D*. *melanogaster*. Following replacement of 1 mM 5-HT with 1 mM ketanserin.
(WMV)

S4 Movie Enhancing effect of 5-HT on isolated crop contraction rate in adult *D*. *melanogaster*. Following replacement of hemolymph with 1 mM 5-HT.
(WMV)

## Abbreviations

mAP: muscle action potential
AKH: adipokinetic hormone
5-HT: serotonin
DILP: *Drosophila* insulin-like peptide
IPC: insulin-producing cell
AC: adenylate cyclase
PKA: protein kinase A
cAMP: cyclic adenosine monophosphate
CREB: cAMP response element binding protein
CC: corpora cardiaca
TAG: thoracico-abdominal ganglion.
